# Health system readiness for innovation scale-up: the experience of community-based distribution of injectable contraceptives in Nigeria

**DOI:** 10.1186/s12913-019-4786-6

**Published:** 2019-12-05

**Authors:** Oluwaseun Akinyemi, Bronwyn Harris, Mary Kawonga

**Affiliations:** 1 Department of Health Policy and Management, College of Medicine, University of Ibadan, Ibadan, Nigeria; 2 Department of Community Health, School of Public Health, Faculty of Health Sciences, University of the Witwatersrand, Johannesburg, South Africa; 3 Centre for Health Policy, School of Public Health, Faculty of Health Sciences, University of the Witwatersrand, Johannesburg, South Africa; 4 Division of Health Sciences, Warwick Medical School, University of Warwick, Coventry, UK

**Keywords:** Scale-up, Community-based distribution of injectable contraceptives, Health system readiness, Stakeholder collaboration

## Abstract

**Background:**

Following the successful pilot of the community-based distribution of injectable contraceptives (CBDIC) by community health extension workers (CHEWs) in Gombe, northern Nigeria in 2010, there was a policy decision to scale-up the innovation to other parts of the country. However, there is limited understanding of health system factors that may facilitate or impede the successful scale-up of this innovation beyond the pilot site. Thus, this study assessed the health system readiness to deliver CBDIC in Nigeria and how this may influence the scale-up process.

**Methods:**

This study was conducted in two Local Government Areas in Gombe State in September 2016. Seven key informant interviews were held with purposively sampled senior officials of the ministries of health at the federal and state levels as well as NGO program managers. Also, 10 in-depth interviews were carried out with health workers. All transcripts were analyzed using the thematic framework analysis approach.

**Result:**

The availability of a policy framework that supports task-shifting and task-sharing, as well as application of evidence from the pilot programme and capacity building programmes for health workers provided a favourable environment for scale-up. Health system challenges for the scale-up process included insufficient community health workers, resistance to the task-shifting policy from professional health groups (who should support the CHEWs), limited funding and poor logistics management which affected commodity distribution and availability. However, there were also a number of health worker innovations which kept the scale-up going. Health workers sometimes used personal resources to make up for logistics failures and poor funding. They often modify the process in order to adapt to the realities on the ground.

**Conclusion:**

This study shows health system weaknesses that may undermine scale-up of CBDIC. The study also highlights what happens when scale-up is narrowly focused on the intervention without considering system context, capacity and readiness. However, agency and discretionary decision-making among frontline health workers facilitated the process of scaling up, although the sustainability of this is questionable. Benefits observed during the pilot may not be realised on a larger scale if health system challenges are not addressed.

## Background

In Nigeria, following the successful pilot of a community-based distribution (CBD) of injectable contraceptives programme in Gombe State (one of the country’s 36 states), a decision was taken by the National Council on Health – Nigeria’s highest decision-making body on health – to scale-up the intervention starting with expansion elsewhere in Gombe State and later extending to other parts of the country [1]. The CBD of injectable contraceptives, which was included in the national task-shifting policy, entails community-based provision of injectable contraceptives by community health extension workers (CHEWs). Scale-up was intended to ensure that benefits identified during the pilot (particularly higher contraceptive uptake) [1] were made available throughout the country.

This innovation was important in Nigeria, a country with very low contraceptive prevalence rates (CPR) - 14.6% for any method and 9.7% for modern methods (condoms, pills, intrauterine devices and implants) [2–4]. In addition, there is a wide dichotomy between contraceptive usage in the urban and rural areas – 24.1 and 10.1%, respectively [5–7]. The 2013 demographic and health survey showed only 3% of married women in Nigeria used an injectable contraceptive method, a total fertility rate of 6 per woman, and an annual population growth rate of 3.2% [7]. The low contraceptive prevalence is said to be an important contributor to the unacceptably high maternal mortality ratio of 576 per 100,000 live births with maternal deaths accounting for 32% of all deaths among women aged 15–49 years [7].

In 2014, scale-up of the CBD of injectable contraceptives, started in Nigeria with a national NGO leading the process. Scale-up started with the training of trainers (experienced nurse midwives) as well as Community Health Extension Workers (CHEWs) [8]. By end of 2017, large scale provision of injectable contraceptives at the community level had commenced in Gombe State (North East) and Kebbi State (North West). However, the understanding of health system factors that may facilitate or impede the successful scale-up of this innovation beyond the initial pilot site is limited.

### Health system and innovation scale-up in low and middle-income countries

The importance of scaling up health interventions in order to maximise population-wide benefits has been well documented [9, 10]. Health innovation scale-up is imperative in order to achieve universal health coverage and global development goals [11–13]. Scaling up can be described in several ways but is generally agreed to mean improving the scope and magnitude of health interventions in order to reach more people in terms of number and geographical spread [9, 10, 14]. Scaling up requires commitment in terms of political will, finances, human and material resources [10, 15]. However, in Low and Middle Income Countries (LMICs), there is a dearth of enabling policies to support scale-up of tested interventions [16]. As alluded to by Gilson and Schneider [13], political will and a conducive policy framework are essential to successful innovation scale-up. Furthermore, sometimes scale-up is embarked upon without a thorough appraisal of health system readiness to deliver the innovations [17], resulting in limited availability of interventions of known efficacy in many low-income countries [1, 18].

The strength and absorptive capabilities of the health system affect scale-up [10, 19]. Health system factors including weak leadership and governance, inadequate human resources for health [20, 21], limited financing [11], the lack of a clear legal framework, over-centralized health systems [22] and weak stakeholder advocacy [13, 23] limit the ability of the system to utilise increased aid inflow [10] and to implement interventions on a wide scale [21]. It has been demonstrated that assessing the health system’s readiness and preparing the system to implement innovations on a large-scale is critical for successful realisation of the expected health benefits of the innovation [17]. According to Evans and Etienne [24], increasing resources will not necessarily lead to increase output in a milieu of health system fragility. This is particularly so in Nigeria, a country with a complex and weak health system [25]. According to the AIDED model of Bradley and colleagues [16], assessing the system context in which an innovation will be delivered is one of the key stages in the process of scaling up an intervention. The AIDED model conceptualises scale-up as a set of five interconnected and non-linear stages: assess, innovate, develop, engage and devolve. The Assess stage includes understanding the context (including socio-cultural and health system context) in which the innovation will be introduced [16]. An assessment of health system readiness to scale-up the community-based delivery (CBD) of injectable contraception has not been documented in Nigeria where a policy decision was taken to scale-up the CBD innovation [1].

This study is part of a broader research project applying the AIDED model [16] to explore possible barriers and facilitators of the scale-up of CBD of injectables in Nigeria.

This paper specifically explores factors in the “assess” component of the AIDED model. The paper aims to identify factors within the health system context that may support or resist the scale-up of the community-based distribution of injectable contraceptives beyond Gombe State, and to explore whether the readiness and receptivity of the health system context was considered in the process of scaling up.

## Methods

### Study design and setting

This study, which was part of a larger study to explore the scale-up of community-based injectable contraceptives in Gombe Nigeria, was a cross-sectional qualitative study comprising key informant interviews (KIIs) and in-depth interviews (IDIs). The study took place principally in Gombe state (North eastern Nigeria) although some interviews took place in Ibadan in the South West (where the national NGO that spear-headed the pilot is headquartered) and Abuja in the Federal Capital Territory (where the Federal Ministry of Health and the international NGO are based). Administratively, Gombe is divided into 11 Local Government Areas (LGAs). Each LGA is further sub-divided into administrative wards. The state has a population of 2,353,879 people according to the 2006 population census of which women constitute about half (49.9%) and women of child bearing age about 22%. The maternal mortality rate is 1726/100,000, one of the highest in the country, and contraceptive prevalence rates (CPRs) are 3.5% for modern methods and 4.0% for any method [1, 3], compared to a national CPR of 15.1% (for any method) [26]. Prior to the adoption of the task-shifting policy which introduced the community-based distribution approach, administration of injectable contraceptives was facility-based and done by nurses and doctors at all levels of health care delivery [1]. Under the new policy thrust, the injectable contraceptives are administered at the community level by CHEWs who are linked to health facilities. Doctors and nurses based in the health facilities support and supervise the CHEWs and manage any adverse effects [1].

### Sampling and participants

Two LGAs in Gombe State – Gombe LGA (urban) and Yamaltu/Deba LGA (rural) – were selected for this study. These study sites were purposively selected because Yamaltu/Deba LGA was one of the sites used for the pilot of the innovation and Gombe LGA because it is the capital of the State and the seat of policy makers at the state level. Study participants were selected primarily from these two LGAs, but also from the State and Federal (national) levels (Table 1). Seventeen participants were recruited through purposive sampling and included health managers in the state and federal Ministry of Health (MoH), as well as programme managers of Non-Governmental Organizations (NGOs) in Ibadan and Abuja who were involved in the pilot study or in making decisions about the scale-up. Other participants included health workers (doctors, nurses and CHEWs) in Gombe (see Table 1).

**Table 1:** Study sites, participants and data collection method

Location	Data collection method	n	Type of participants
IBADAN (state level)			
	KII	1	NGO program manager (doctor)
ABUJA (national level)			
	KII	3	NGO program managers, family planning coordinator of the Federal MoH^a^ (all doctors).
GOMBE			
Gombe LGA	KII	3	State family planning coordinator, Director PHC^b^, Senior official of State MoH.
	IDI	5	Health workers (Nurses, CHEWs^c^)
Yamaltu/Deba LGA	IDI	5	Health workers (CHEWs^c^, Nurses)
Total		17	

^a^MoH – Ministry of Health; ^b^PHC – Primary Health Care; ^c^CHEWs – Community Health Extension Workers

### Data collection and analysis

A total of seven KIIs and 10 IDIs were conducted with the study participants over a two-week period in September 2016 to explore different roles and perspectives from across the health system hierarchy – both those responsible for managing the scale-up and those actually delivering the innovation. Interviews were conducted in English, audio recorded and transcribed. KIIs were conducted using a KII guide (see Additional file 1) to explore participants’ roles in implementation of the CBDIC intervention during and after the pilot, their understanding of the task-shifting policy, as well as perspectives of challenges and factors supportive of the scale-up of CBDIC within the health system context. Also, IDIs were conducted with health workers using an interview guide (see Additional file 1) to assess their roles and experiences in the wider implementation of the CBDIC innovation, and their perspective of enablers and barriers.

A broad coding framework was developed based on the research questions, and applied to the transcripts to identify emerging and divergent themes. All transcripts were analyzed with NVIVO (version 10) software using the thematic framework analysis approach [27, 28]. As themes emerged, these were indexed and compared with themes from subsequent interviews until a sense of attainment of saturation was achieved [27].

## Results

### Factors in the health system context that may impede scale-up

Participants described a number of factors in the policy and health systems context which may impede the scale-up process. These factors include human resource and operational challenges as well as resistance to task-shifting from health professional bodies.

#### Human resource and personnel training challenges

The scale-up plan was to extend the service to reach all administrative wards in all LGAs in Gombe State. Shortages of human resources to cover all Gombe’s administrative wards was a major drawback for the community-based delivery of injectable contraceptives. This led to many clients being unable to use contraceptives consistently.

“…*the challenges we encountered was lack of adequately trained personnel because it was limited at least one person per ward, one CHEW will go into the ward and then, the woman will patronize her (the CHEW) … so there was inadequate trained staff*” (KII5, male, family planning coordinator).

Furthermore, participants were of the opinion that the task shifting policy adopted by the federal government about six years earlier was not adequate to address the human resource to deliver the service. They were of the opinion that allowing other cadres of health workers (like pharmacy assistants, radiology assistants, environmental assistants and community pharmacists) apart from nurses and CHEWs to administer the community-based injectable contraceptives would accelerate scale-up since the commodities would be more readily available in the community.

“…*the federal government should include all the health workers and not limit (task-sharing of CBD of injectable contraceptives) to only midwife or nurse or even the CHEWs in the community. These are the ones nearer to their communities in terms of training, in terms of updating their (community’s) knowledge. So, we need to carry along all health professionals so that we’ll have a wider coverage …let’s carry them together, give them training, update their knowledge and build their capacities, they can do it*” (IDI2, female, CHEW).

A health manager at the local government level described having to do a lot of administrative duties within a very limited time, thus working under a lot of pressure in order to conform to the NGO requirement. Thus, managers were also under a lot of pressure and not able to adequately play oversight of the programme nor achieve their targets and goals. According to a manager, “…*they (coordinating NGO) will send about one-year report for us to write in just two days we will have to crash the program, crash the work to send them message*…” (IDI9, male, Deputy PHC Coordinator).

In addition, in terms of training, early implementers of the scale-up (the national and international NGOs coordinating scale-up) reported challenges with developing tools to match the capacity level / training needs of lay health workers like CHEWs. This slowed down the scale-up process, as implementers could not get as much done within time planned.

“…*Our challenges had to do with providing simple competency-based tools – you know traditionally, the training manuals tends to be very voluminous, then too wordy – you just need practical skills immediately, how do you provide an injection*?” (KII3, female, NGO program manager).

#### Weaknesses in service delivery support systems

Other challenges which were thought to have hampered the scale-up process included lack of transportation for health workers to get to specific communities and poor logistics management. This affected commodity distribution and availability and hence limited the number of women they could reach with the intervention.

“*The problem is nothing but transport …if there is transport, enough transport, we can go out and you see like home visit, outreach, (but) if there is no transport, there’s problem. But if there is transport, we can go out at least twice in a week, we can find the women at home nursing there and they can accept it (the injectable contraceptive)*. (IDI14, female, CHEW).

“…*if there is break in the chain of the supply then there will be a problem, but if there is a continuous supply of these injectable then I don’t think there will be any problem”* (IDI9, male, Deputy PHC coordinator).

Also, the centralised supply chain system was a barrier to effective distribution of the commodity to the community level as availability of transportation was a rate-limiting step in the scale-up process. Contraceptives are usually imported into the country by donors and kept in the country’s central store in Lagos. Donors also fund the distribution of the commodity from Lagos to the states. However, the bottleneck is getting the commodity from the state capitals to health facilities, the so-called “last mile delivery” due to poor funding of logistics by the states. According to a key informant, this has adversely affected contraceptive security in many states in Nigeria.

“*Another challenge was commodity security so at that point in time CLMS, that’s community logistics management system, had issues in terms of moving commodities from the federal level to the state level and down to the facility level*…” (KII3, female, NGO program manager).

#### Commodity security

In addition, participants reported that poor logistics management made the product vulnerable to theft. One key informant explained that there were pilferages along the logistic chain such that injectable contraceptives were diverted into private patent medicine stores and sold unlike in the public sector where they are freely available. According to the key informant, *“Contraceptives disappear from the logistics chain into the private sector because they (contraceptives) are totally free in the public sector. How this happens is not clear”* (KII3, female, NGO program manager).

#### Resistance to the task-shifting and task-sharing policy from health professional groups

An important reported health system challenge to the scale-up was persistent conflict among health worker groups about which cadre of health worker should administer injectable contraceptives.

“…*there is a perennial struggle within (the health sector) about whose domain is injectable contraceptives. While the community health workers feel they should be in charge, nurses will always feel that they are the people giving it, and should continue to give it*” (IDI, male, Doctor, SMoH).

Furthermore, one of the program implementers narrated that health workers and professional health groups at the onset of both the pilot and the scale-up were not supportive of CHEWs delivering injectables. Some professional bodies were particularly concerned about poor safety and quality if CHEWs were allowed to administer injectable contraceptives. For example, according to the Medical and Dental Council of Nigeria, *“…their (CHEWs’) basic training was not enough (for them to administer injectable contraceptives)”* (KII3, female, NGO program manager). This sentiment was reportedly shared by other professional associations particularly the Nursing Council as shown by the quotes below:

“…*many of the medical associations and professional bodies were a bit reluctant, the Nursing Council particularly of note, was quite uncomfortable with training that cadre of staff (CHEWs)*. (KII1, male, NGO program manager).

“…*there was serious resistance from them (Nursing Council). Also, a few other professional bodies raised some concerns as to the quality of services especially when it relates to needle or injection safet*y” (KII4, male, FMOH official).

#### Lack of ownership of the scale-up process at the state and local government levels

The states and the local government health authorities were reportedly unwilling to drive the initiative to scale-up CBDIC. Participants perceived that this apathy was largely caused by the states’ poor funding support to implement the CBDIC. Many of the state MOHs had line items in their annual budget to fund the introduction of injectables, but had no financial support from the federal level to accomplish such plans. Furthermore, there was usually limited ownership from the State and Local Government tiers of governance when the NGOs are perceived to be the drivers of the initiative.

Most of the states are almost completely dependent on NGO partners for sustenance and funding in many areas that are not funded by government leadership including CBDIC. The Federal Ministry of Health usually supervises the State MoHs, which in turn oversee the Local Government health authorities in their respective states.

“…*So they (the federal government) are fully involved from the outset… they lead as far as the implementation is concerned, so what we (the partners) do is get in the back seat and try to give technical support for the state to be able to implement such activity…The government can easily talk to them (the community) than what a partner will do when such a challenge comes.*” (KII1, male, NGO manager).

#### Ineffective monitoring and evaluation

Participants reported the lack of effective monitoring and evaluation of health workers’ activities during the scale-up process as an important factor affecting smooth scale-up. An interviewee alluded to the fact that there might be a discordance between the report submitted by some implementers and the realities on the field:

“… *there is need also even from the state, from the LGA to follow-up, see how things are being done, are they really doing the right thing? Are they really delivering the services not just bringing results on paper? When you go (to the field) the results may not be there vividly. There should be a level of follow-up, monitor them to see how they are going on. Even (members of) the community, interview them- how are the services, are you satisfied with what is happening?*” (IDI2, female, CHO).

### Factors in the health system context that may facilitate scale-up

#### Enabling task-shifting policy

Some key informants were of the opinion that availability of a policy framework which supported task-shifting of injectable contraceptives to CHEWs was fundamental to the government support for the innovation scale-up. Also, the task-shifting policy was vital for the political will from the top (the federal government) through to the Local Government Areas. In the view of a respondent:

“*I think the major factor (in successful implementation) is the conducive environment that the policy on task-shifting created …this brought an overwhelming support of the government of Nigeria toward the policy implementation*” (KII1, male, NGO manager).

However, the lack of funding transfers from federal government for the new programme reflected that the other element of political will – resource allocation – was limited.

#### Collaboration between implementing NGOs and the government

From the outset of the implementation process, the coordinating NGOs yielded the leadership of the programme to the Federal Ministry of Health in order to overcome resistance and to guarantee cooperation from the States and the local governments. When states saw that the federal government was in the lead, resistance fell away.

“…*When we go to implement a project, we want to be sure that the state government has a kind of ownership of the process… Once the FMOH gave a clear nod to the project, there was limited resistance from the states, as the project had an official introductory note that facilitated the engagement of the actors at the local level. The state was able to address the pockets of resistance wherever they arose by referring to the national support for the program.* (KII1, male, NGO manager).

#### Continuous stakeholder collaboration

Key informants also narrated that resistance was constantly dealt with through continuous dialogue and stake holder participation during the pilot and the scale-up process.

“…*At the outset, there was a stakeholder meeting that involved many of the professional bodies, so it was easy to always go back to them whenever you have a challenge*” (KII1, male, NGO manager). Another participant added: “*I think through dialogue with the various bodies it (injectable contraceptive by CHEWs) was finally allowed*” (KII3, female, NGO manager).

#### Quality assurance measures to prepare for innovation scale-up

Training and capacity building programmes for health workers aided the scale-up process. In the opinion of a participant, *“…with the training and capacity building (given to health workers), I think it showed a lot of result in both pilot and scale-up - over 11,000 providers were trained by both UNFPA and DKT Nigeria (a social marketing company) between 2015 to 2017..*.” (KII1, male, NGO manager).

In addition, quality control measures were put in place before scale-up. These included provision of adequate training for CHEWs and initiation of the injectable contraceptives at the health facility (so that health professionals could screen users for possible contra-indications) before allowing CHEWs to continue with subsequent monthly provision in the community. At the community level, contraindications or side effects that develop during use were reported to the community-based distributors who were usually in close proximity to the users. The community-based distributors were given a pictorial adaptation of the medical eligibility criteria chart which was used for counselling and screening at the community level.

*“… So, most of our trainings are focused on quality of service and by this I mean, the community-based groups are more positioned to do harm when they are not properly trained and that will kill the whole process from the start. So, we’ve paid lot of attention to looking at eligibility of women. Both the community-based groups (pharmacy technicians, community pharmacist, community volunteers as well as retired CHEWs and midwives) and the CHEWs were taken through intensive training on medical eligibility for hormonal contraceptives using the WHO Medical Eligibility Criteria (MEC)…The MEC has been adapted in pictorial form for ease of use by CHEWs at the community level, so they are able to detect contraindications, counsel and refer appropriately*” (KII1, male, NGO manager).

“*Initiation is done at the facility level but refills or subsequent monthly injectables just like DMPA-SC (subcutaneous Depot Medroxyprogesterone Acetate) is done by them [CHEWs]. We were able to allay a lot of anxieties that ‘women, who are hypertensive, how do they check their BP?’ And all of those things*” (KII1, male, NGO manager).

CHEWs also had a one-week training in contraceptives technology and counselling. This included clinical practicum sessions, didactic lectures, value clarification and rights-based decision making.

Furthermore, contraceptive products and consumables were kept in bags called outreach kits, designed to keep products at a stable temperature. The community-based distributors were also advised to keep products in an airy space out of the reach of children. Likewise, another quality control measure was to develop an effective referral system and linkages with hospitals that could take care of possible side effects. These measures helped to allay the fears of concerned groups, particularly the professional bodies, about the safety of the innovation.

*“As part of our quality control measures we have a one-week intensive training for CHEWs in contraceptive technology and counselling which encompassed practical training sessions in hospitals, role plays and didactic lectures, rights-based decision making and value clarification in order to ensure that the woman knows her rights and is not railroaded into adopting a contraceptive method”* (KII1, male, NGO manager).

“*The other thing we have done is to do a complete community-to-hospital continuum of service/linkage that will ensure effective referrals. At the start-up of the project, the first few months, we insisted that women who were seen, who wanted the method can be counselled, given advice and must be referred to the facility to have a proper assessment done*” (KII1, male, NGO manager).

*“At the PHC facility, contraceptives are stored like other medicines but we designed a special bag called outreach kit for community-based distributors in order to keep contraceptives and other consumables within a stable temperature. Community-based distributors are also trained to store products properly out of the reach of children and to dispose waste safely”* (KII1, male, NGO manager).

### Action taken by implementers to address system weaknesses

#### Innovation and agency of health workers

Despite the transport and supply chain management barriers, the innovation and agency of health workers enabled service provision to continue. For example, health workers (CHEWs) sometimes spent personal funds on transportation in order to provide services to people in their communities. Also, in order to get to the hard-to-reach areas, health workers took the initiative to reschedule most outreach activities to the dry seasons when it is much easier to access those terrains. Usually, the nurses plan the outreaches, supervise the CHEWs and they usually stay at the facilities to provide services whereas the CHEWs do the community delivery of the injectable contraceptives.

The limitation of this initiative however is that the women in these hard-to-reach areas may not be able to access injectable contraceptives during the rainy season and are then unprotected during this period or they may use other substitutes like oral pills or condoms. However, family planning managers are working on making longer-acting contraceptives (for example Sayana Press given once in three months) more available and to empower women to self-administer these.

“*They (CHEWs) are going (on outreaches) with NAPEP (tricycle) and the motorcycle…with their money*” (IDI14, female, Nurse).

“*We plan most of our [far out] outings for the dry season and during raining season we go to nearby places*…” (IDI11, female, Nurse).

*“The truth is that during the rainy season the woman in the hard-to-reach area may get pregnant as she might not be able to get the injectable contraceptives. This is one of the challenges of community-based distribution in Nigeria – poor access to care during rainy season. We plan to deal with this challenge by popularising longer acting contraceptives, specifically Sayana Press, and training providers who will train women to self-inject these longer acting contraceptives.”* (KII1, male, NGO manager).

Additionally, health workers, particularly CHEWs dealt with the challenge of human resource shortages by operating from home instead of going door-to-door and having the women in the community come to the CHEW’s house to access injectable contraceptives services. This approach helped CHEWs to reach many more women despite the challenges with transportation. According to a key informant, “…*the woman will patronize her (a CHEW) at home [because] there was inadequate trained staff*” (KII5, male, family planning coordinator).

## Discussion

In this study, we collected qualitative data on health system factors that may influence the scale-up of community-based injectable contraceptives in Gombe State, Nigeria. We also examined how health system challenges were dealt with within the context of the research setting. Our discussion addresses the health system concerns and implications for further scale-up of the innovation.

Principal among factors facilitating the scale-up process was political commitment by the federal government of Nigeria. Also, the availability of a policy framework supporting task shifting of administration of injectable contraceptives from the doctors and nurses to allied health professionals like the CHEWs is another important aspect of the political commitment. However, although shifting of roles to CHEWs was necessary for a successful scale-up, it was not sufficient as the task shifting policy was rather generic and does not cover other pertinent issues like commodity supply, logistics and funding. Therefore, the lack of policy and policy implementation guidelines on how to implement the innovation in Nigeria’s health system context may explain the seeming inadequate preparedness with regards to funding, workforce planning and logistics management in the process of scale-up. According to Berlan and colleagues [29], policy formulation and proper implementation is imperative in the process of innovation diffusion. The constraints to scale-up identified in this study include shortages in human resource, poor logistics management as well as theft of commodities, resistance to the task-shifting policy from health professional groups and lack of ownership of the process by the state and local government.

The availability of a policy framework is closely associated with the political leadership. In the work of Mangham and Hanson [10] and others [13], political will and availability of a conducive policy framework were shown to be essential to successful innovation scale-up. Strong political leadership is said to be critical to the realisation of the global developmental goals [13]. Evidence abounds that a pilot project is essential in documenting best practices, creating an enabling environment for a health innovation, enhancing the quality and improving the capacity of health workers to deliver the innovation [1]. And this was borne out in this scale-up.

Findings from this study show that despite an enabling task-shifting policy, there are still a number of barriers relating to funding, which may impede scale-up. Issues impeding the process particularly relate to inadequate transport for CHEWs, and logistical problems with commodity distribution. Thus, there seems to be a focus on the innovation but inattention to the delivery system. In a study done in Swaziland, on scale-up of voluntary medical male circumcision, Edgil and colleagues demonstrated that sound supply chain management is pivotal to the success of wide scale implementation of a health innovation and that it is imperative for program managers to plan and budget for this from the beginning of the process [30]. Although, the street level bureaucrats in our study demonstrated resilience and ingenuity in the face of failure of logistics to further implementation of the policy provisions, nevertheless the sustainability of their stopgap measures is questionable. Studies have shown that frontline health workers can exercise considerable discretionary powers thus making them important determinants in the implementation of policies [31, 32]. However, how much discretion these street level bureaucrats should be allowed during implementation is still a subject of debate [33]. Furthermore, in the Nigerian context where people are already stretched by high out-of-pocket expenditure on health [34], requiring that they buy injectable contraceptives, when these are not available through public facilities and channels of distribution, undermines the aim of increasing contraceptive prevalence. Political leadership (particularly as expressed through financial commitment and allocation) is needed in this context, but it is not inherently spontaneous, and rather comes through continuous advocacy and government engagements by stakeholders [13, 23]. Advocacy for improved health funding should however not leave out the private sector. The role of the private sector has been described as “large and important” in global health funding [35]. Furthermore, there is increased need for advocacy to translate the current political will into greater financial commitment and increased funding of the scale-up process. According to Gilson and Schneider, an advocacy drive should be focused and targeted at both the political leadership as well the public through lobbying in order to frame public opinion and set the agenda for policy makers [13].

Findings from the study show that insufficient human resources for the scale-up contributed to overwork for the few health workers available, despite the implementation of the task-shifting policy of the federal government. Although the task-shifting policy is officially being implemented in the country, its implementation is still sub-optimal, possibly due to the passive resistance of higher cadre health workers to the process, at least at the inception. In a Malawian study to assess the human resource requirement for the scale-up of highly active antiretroviral therapy, the researchers concluded that the process is labour-intensive and that there cannot be successful scale-up without a commensurate increase in the human resources for health [36]. Thus, inadequate human resource may put significant stress on health workers who are the most important resource of the health system [37]. Scaling up health innovations in a milieu of human resource inadequacy may result in neglect of some other critical health services [38], as well as poor health service provision to citizens [39]. Similarly, in a systematic review of health systems bottlenecks to essential maternal and newborn healthcare, Dickson and colleagues [21] submitted that the principal health system blockage to scale-up of health innovations were human resource for health issues (workforce forecasting, task sharing, motivation for health workers in rural areas), limited funding, challenges in the accessibility of healthcare and unavailability of essential medical products and technologies. Further research is needed to estimate the human resource need for the scale-up of injectable contraceptives in Nigeria and possibly to assess the stress on the available health workers from the current scale-up [36, 38].

Studies have established the contributions of community health workers to increasing access to healthcare and health innovations, reducing cost of care, improving the quality of care as well as contributing to community growth and empowerment in poor and underserved areas [40–42]. Although health workers in this study exercised agency to make sure the commodity gets to women, but their motivation may wane in the face of lack of ‘secure funding’ which may make the scale-up program unsustainable [40].

Furthermore, program managers and policy makers should make improved budgetary provision for supply chain management [30] in the scale-up of the community-based provision of injectable contraceptives and the budget should be monitored for compliance. Likewise, practical procurement and logistics management systems should be instituted in order to ensure constant availability of contraceptive products and delays in the system should be investigated and sanctioned appropriately [21].

Also, there may be deliberate policies to attract, deploy and retain CHEWs and other health workers in the rural areas as well as to stem the tide of in- and out-migration [37]. Increasing the retirement age of health workers may also be considered [21]. Besides, participants in our study described conflicts among professional health groups around role and task-shifting which slowed down the scale-up process. However, this type of inter-professional conflict, where professions seek to guard their perceived jurisdictions, is not peculiar to Nigeria [43]. Hall [44] believes that more education for health workers about other professional groups’ ideals, philosophies, customs and conducts may help improve cooperative practice among health professional groups. Organizing interdisciplinary discourse and continuing education for professional groups aimed at enlightening health workers about the roles, values, history and cultures of lay health workers may help to limit conflicts among health worker groups [43–46]. This sort of team-building intervention should be proactively done before innovation implementation and scale-up rather than being reactive when resistance might be more difficult to deal with as seen in this study.

The findings of this study revealed some challenges in the first phase (the ‘assess’ phase) of the scaling up process of community-based distribution of injectable contraceptives in Nigeria [18]. Since the stages of the AIDED scale-up model are interconnected and non-linear, there may be adverse implications for other stages of the scale-up process if the context in which the innovation will be introduced is not properly assessed and prepared. Thus, this study points to some of the issues that could be considered in Nigeria before scaling up the community-based injectable contraceptives to more states.

### Study limitation

Certain limitations of this study should be highlighted: findings may not be generalizable to other local contexts in Nigeria as the country is very diverse. Nonetheless, the findings of this study provide useful insights into understanding how health systems readiness affect the scale-up of health innovations in a developing country like Nigeria.

## Conclusion

This study highlights that although a task-shifting policy exists to facilitate the scale-up of community-based distribution of injectable contraceptives by CHEWs, health system challenges may impede scaling up of this innovation. In our study setting, the health system needs more readying, which includes encouraging interdisciplinary discourse in order to help reduce or prevent health worker resistance to the task-shifting policy and to promote industrial harmony among health worker groups. Also, support systems for innovation scale-up require more attention – human resource, supply of commodities and resource allocation. A key implication for weak health system contexts like Nigeria is that inattention to readying the health system may undermine the scale-up of innovations of proven effectiveness.

## Additional file

**Additional file 1.** Interview Guide 1: Key informant interview with senior MOH officials and NGO programme managers. Interview guide 2: In-depth interview with health workers (Doctors and CHEWs).

## Data Availability

The data (transcripts) used and/or analysed in this study are available from Dr. Oluwaseun Akinyemi on request.

## Abbreviations

AIDED: Assess Innovate Develop Engage Devolve
CBD: Community-based delivery
CBDIC: Community-based distribution of injectable contraceptives
CHEW: Community health extension worker
CHO: Community health officer
CPR: Contraceptive prevalence rates
IDI: In-depth interview
KII: Key informant interview
LGA: Local Government Authority
NGO: Non-governmental organization
PHC: Primary Health Care
