# Supplementary material for: Health system readiness for innovation scale-up: the experience of community-based distribution of injectable contraceptives in Nigeria
Source: BMC Health Serv Res. 2019 Dec 5;19:938. doi: 10.1186/s12913-019-4786-6 (PMC6896335; doi:10.1186/s12913-019-4786-6)
Supplement: Supplementary file 1 — Additional file 1. Interview Guide 1: Key informant interview with senior MOH officials and NGO programme managers. Interview guide 2: In-depth interview with health workers (Doctors and CHEWs). [file 12913_2019_4786_MOESM1_ESM.docx]

## **Interview Guide 1: Key informant interview with senior MOH officials and NGO programme managers**

1. Let’s start by you describing your role in implementing this intervention – the community-based delivery of injectable contraceptives in the pilot sites in Gombe. What was your role and how long were you involved?
2. Did you encounter any challenges during the pilot study? Please explain.

***Probes:***

- If not mentioned, ask: any resistance from the community members, from health workers, from other stakeholders, from potential users themselves?
- How did you handle challenges / resistance?

1. We are interested in your experience with the process of taking the intervention from pilot study phase to wider implementation in Gombe itself. What was the process, from implementation to wider scale up of the intervention? Walk me through the process.

***Probes:***

- The goal, approach to implementing, key components of the process, indicators of success
- Landscape and stakeholder assessment done – before, during or after the pilot?
- Your role in these processes?

1. In your opinion, what factors have supported the scale up of this intervention so far?
2. Have you experienced challenges during the process of scaling up? Please explain.

***Probes:***

- What challenges? ... ***may prompt with*** “any others”
- How did you handle challenges?

1. Was there a way of ensuring that the intervention aligns to the wants and preferences of the users you were targeting? Please tell me about it.

***Probes:***

- Any user needs / wants assessment done? By whom? When?
- Any modifications made in order to align packaging of the innovation to user wants – either during or after the pilot?
- Any health service modification (e.g. change service models for delivering the intervention)?

1. Have communities been engaged in the scale up process? Please describe in what ways and by whom?
2. Looking back, is there anything that you think could have been done differently to facilitate the process from pilot study to wider implementation? Please explain.
3. Is there anything else we have not asked but you would like to tell us about your experience with the process of implementation and scale-up of CBD?

**Thank you for your time. This is the end of our discussion.**

**Interview guide 2: In-depth interview with health workers (Doctors and CHEWs)**

1. Let’s start by you describing your role in implementing this intervention – the community-based delivery of injectable contraceptives by community health extension workers.
   1. What role did doctors / CHEWs play in designing the intervention?
   2. What about their role in introducing the intervention at community level?
   3. What was your own role?

***Probes:***

- **Doctors:** Your own (or experience of other doctors like you) with: planning, training CHEWs, providing supportive supervision to CHEWs during and after the pilot?
- **CHEWs**: Your own (or experience of other CHEWs like you) in planning, administering the injectable contraceptives to women?
  1. Did you / other health workers experience challenges?
     1. Please explain.
     2. How did you handle the challenges?

1. In your opinion, what factors affect wider implementation and uptake of the CBD of injectable contraceptives in the communities that you serve?

***Probes:***

- Anything else?

1. In your opinion, how supportive of this innovation are:
   1. **For doctors**: doctors in general, and your professional health group (e.g. Nigerian Medical Association) please explain.
   2. **For CHEWs**: community health extension workers in general? Please explain.
2. As far as you are aware, what role, if any, have health workers like you played in making the CBD of injectable contraceptives more acceptable to the users in your community? Please explain.
3. In your opinion, how likely are those women who have already adopted the CBD of injectables to spread information about the innovation to other women who are not using it? Please explain.
4. Looking back, is there anything that you feel might have been done differently to facilitate the process of moving from pilot study to wider implementation?
5. Is there anything else we have not discussed that you would like to tell us about your experience with implementing the CBD of injectable contraceptives?

**Thank you for your time. This is the end of our discussion.**
